# Dual-layer spectral computed tomography: measuring relative electron density

**DOI:** 10.1186/s41747-018-0051-8

**Published:** 2018-08-22

**Authors:** Kai Mei, Sebastian Ehn, Markus Oechsner, Felix K. Kopp, Daniela Pfeiffer, Alexander A. Fingerle, Franz Pfeiffer, Stephanie E. Combs, Jan J. Wilkens, Ernst J. Rummeny, Peter B. Noël

**Affiliations:** 1 Department of Diagnostic and Interventional Radiology, Klinikum rechts der Isar, Technical University of Munich, Munich, Germany; 2 Department of Physics and Munich School of BioEngineering, Technical University of Munich, Munich, Germany; 3 Department of Radiation Oncology, Klinikum rechts der Isar, Technical University of Munich, Munich, Germany

**Keywords:** Absorption (radiation), Dual-layer spectral computed tomography, Electrons, Radiotherapy, Tomography (x-ray, computed)

## Abstract

**Background:**

X-ray and particle radiation therapy planning requires accurate estimation of local electron density within the patient body to calculate dose delivery to tumour regions. We evaluate the feasibility and accuracy of electron density measurement using dual-layer computed tomography (DLCT), a recently introduced dual-energy CT technique.

**Methods:**

Two calibration phantoms were scanned with DLCT and virtual monoenergetic images (VMIs) at 50 keV and 200 keV were generated. We investigated two approaches to obtain relative electron densities from these VMIs: to fit an analytic interaction cross-sectional model and to empirically calibrate a conversion function with one of the phantoms. Knowledge of the emitted x-ray spectrum was not required for the presented work.

**Results:**

The results from both methods were highly correlated to the nominal values (*R* > 0.999). Except for the water and lung inserts, the error was within 1.79% (average 1.53%) for the cross-sectional model and 1.61% (average 0.87%) for the calibrated conversion. Different radiation doses did not have a significant influence on the measurement (*p* = 0.348, 0.167), suggesting that the methods are reproducible. Further, we applied these methods to routine clinical data.

**Conclusions:**

Our study shows a high validity of electron density estimation based on DLCT, which has potential to improve the procedure and accuracy of measuring electron density in clinical practice.

## Key points

- Dual-layer spectral CT provides accurate electron density estimation (error rate < 1.75%)
- Virtual monoenergetic images from dual-layer CT allow direct calculation of electron densities
- Electron densities generated via dual-energy CT have the potential to improve clinical practice

## Background

Prior to receiving x-ray or particle radiation therapy, it is a crucial step for the patient to undergo computed tomography (CT) so that the optimal dose delivery to the tumour can be calculated while reducing unnecessary radiation exposure to surrounding healthy and critical tissue. This treatment planning is generally achieved by the estimation of the corresponding particle and x-ray absorption properties of the tissue, such as in the form of relative electron density, which is the ratio of electron densities of given materials to the electron density of water.

Conventional single-energy CT can provide a raw estimation of electron density by simply applying its correlations with Hounsfield units (HU), then categorising the values to electron densities [1, 2]. However, this method is not accurate, because conventional HU values, which are defined by the linear attenuation coefficient, also depend on the effective atomic number and the x-ray spectrum. Dual-energy CT (DECT), such as dual-source CT (DSCT) or twin-beam CT, improve this estimation by using two different energy spectra [3]. Various studies have demonstrated the accuracy of measuring electron density using DECT [4–7]. Recently, a detector-based spectral DECT technique, namely dual-layer CT (DLCT), was introduced [8]. This technology allows the acquisition of energy-selective projection data in the two detection layers simultaneously and therefore directly generating spectral information for each CT scan without the need to select specific protocols [8]. Compared with DSCT, DLCT seems to be superior in generating highly accurate virtual monoenergetic images (VMIs) [9, 10] to recognise different materials and to quantitate elemental decompositions, such as intravenous contrast agents or bone mineral density [11–13], which may be of great benefit when estimating electron densities.

There are several ways to approximate electron density. With DECT, the physical interaction cross-sectional model has been applied to dual-energy data [4–7], resulting in a significant improvement in accuracy compared with single-energy CT [3]. However, these methods require exact knowledge of the spectra of the two x-ray beams in the DECT scanner to parameterise the physical interaction mechanisms, so that the accuracy relies largely on the precision of the assumed x-ray spectra. Conversely, calibration methods generate a specific empirical conversion function from measured HU values at two x-ray spectra to relative electron densities by fitting data from known phantoms to their known electron densities [14]. This method does not require beam spectra, but is limited to a specific calibrated scanner model and acquisition protocol.

In this article, we describe both methods, fitting cross-sectional models and phantom-calibrated conversion functions to DLCT. Our aim was to access relative electron densities from VMIs of DLCT and to quantify their accuracy.

## Methods

### CT phantoms, scan protocols and image reconstruction

**Fig. 1:**
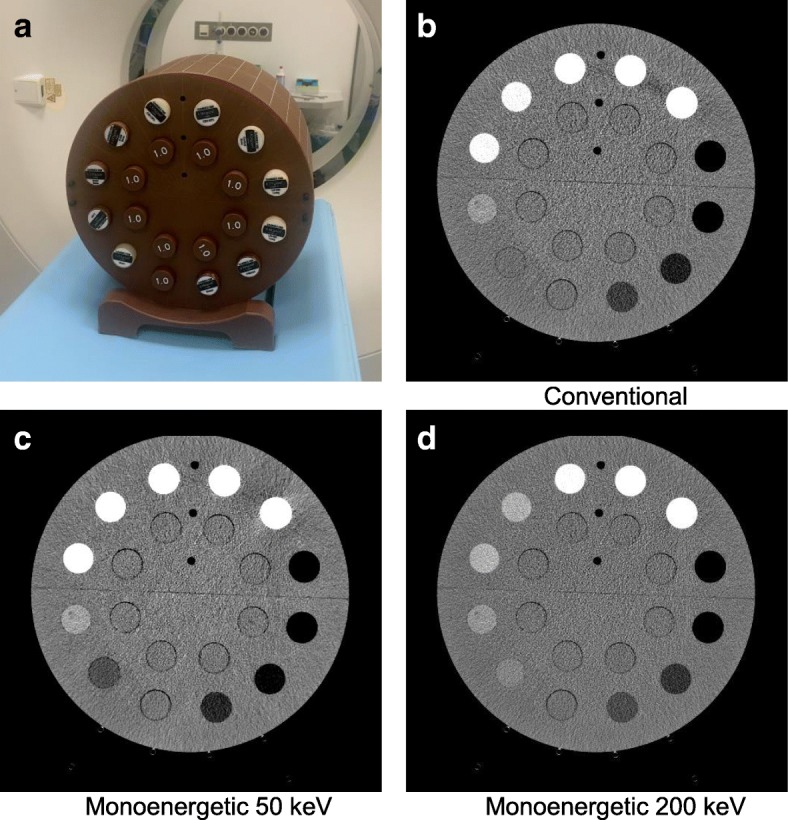
Gammex phantom with inserts simulating human tissue. **a** Photo of the phantom with twelve inserts (relative electron densities ranging from 0.264 to 1.696). **b** Conventional computed tomographic image. **c** and **d** Corresponding virtual mono-energetic images (50 keV and 200 keV). **b**–**d** The window level in these images is 50 HU with width of 350 HU. In the outer ring of the Gammex phantom, the insert with the highest intensity is cortical bone. The electron densities of twelve materials decreases counterclockwise

**Fig. 2:**
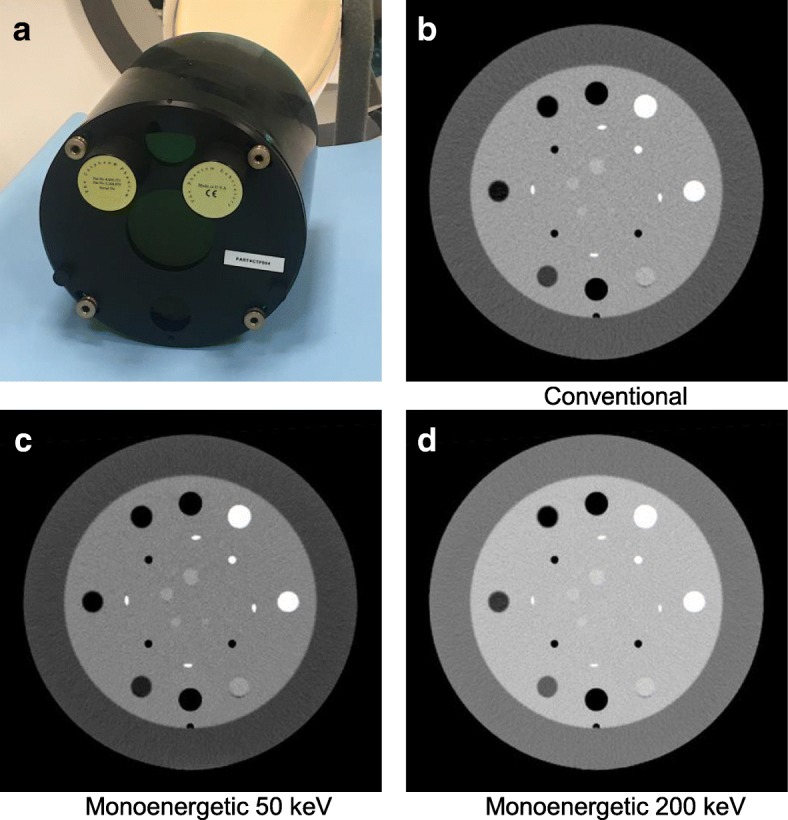
Catphan phantom. **a** Photo of the phantom. **b** Conventional images. **c** and **d** Corresponding virtual mono-energetic images (50 keV and 200 keV). The window level in images **b**-**d** is 50 HU with width of 350 HU. In the outer ring of Catphan phantom (second row), Teflon, Delrin, Acrylic, Polystyrene, low-density polyethylene, and polymethylpentene are located at 1, 3, 5, 7, 9, and 11 o’clock, respectively. Air is located at the 6 and 12 o’clock positions

Two different CT phantoms were used: a Gammex phantom (467-TOMO; Gammex RMI, Middleton WI, USA) (Fig. 1) and a Catphan phantom (Catphan 504; The Phantom Laboratory, Salem NY, USA) (Fig. 2). The Gammex phantom contains 12 inserts representing tissue-equivalent materials with various known electron densities. Each insert is cylindrical, with a diameter of 30 mm and a length of 70 mm. The Catphan phantom has six homogeneous regions consisting of common materials with known electron densities; each region is a cylinder with a 12.5-mm diameter and a length of 25 mm.

Scans were conducted with a DLCT unit, the IQon Spectral CT (Philips Healthcare, Best, the Netherlands). Similarly to the acquisition protocols applied in clinical routine, Gammex and Catphan phantoms were scanned with an x-ray source voltage of 120 peak kilovoltage (kVp), a collimation width of 0.625 mm, a revolution time of 1.5 s and a spiral pitch factor of 0.983. We made four levels of x-ray tube currents, which were 229, 153, 77 and 56 mA, resulting in x-ray exposures of 350, 234, 117 and 86 mA, respectively, and the corresponding volume CT dose index (CTDI_vol_) of 30, 20, 10 and 7.5 mGy was recorded in the dose reports.

The spectral data were reconstructed with an iterative reconstruction at vendor-specific level 2 and a standard filter B, according to the settings used in most clinical abdominal examinations. The field of view was 360 mm, and the slice thickness was 0.8 mm. Corresponding VMIs were generated by using vendor-specific spectral software (IntelliSpace Portal v10.1; Philips Healthcare) at 50 and 200 keV. We used these two monoenergetic levels because at 50 keV the photoelectric effect and at 200 keV the Compton effect are the dominant x-ray interactions with matter. Regions of interest were drawn as cylinders with half of the radius and height of the actual insert dimension, and the mean HU values were measured. Regions of interest were synchronised between VMIs, and the measurements were repeated using image-processing software (ImageJ v1.50f; National Institutes of Health, Bethesda, MD, USA) [15].

### Electron density estimation using cross-sectional model

In order to obtain electron density values using the cross-sectional model, the energy-dependent CT numbers HU(*E*) in the corresponding VMI were firstly converted into the mass-attenuation coefficients *μ*(*E*)/*ρ* at the specific energy using the following equation:

1 $$ \frac{\mu (E)}{\rho }=\frac{\mu_w(E)}{\rho_w}\left(\frac{\mathrm{HU}(E)}{1000}+1\right), $$

where *μ*_*w*_(*E*)/*ρ*_*w*_ is constant representing mass-attenuation coefficient of water at energy *E*, which can be referenced from the National Institute of Standards and Technology database [16].

In an ideal case of a narrow beam of monoenergetic photons in the range of clinical CT (*E* < 511 keV), the mass-attenuation coefficient *μ*(*E*)/*ρ* can be attributed to three physical interaction mechanisms: photoelectric absorption, incoherent (Compton) scattering and coherent (Rayleigh) scattering. For the energy range used in clinical CT, coherent scattering can often be neglected for standard body tissues, resulting in the well-known two-dimensional parameterisation for the mass-attenuation coefficient [17, 18]:

2 $$ \frac{\mu (E)}{\rho}\cong {a}_p{f}_p(E)+{a}_c{f}_c(E), $$

where *a*_*p*_ and *a*_*c*_ are characteristic parameters for the different materials in the image. *f*_*p*_ and *f*_*c*_ are the energy dependencies of photoelectric absorption and Compton scattering. The photoelectric absorption part is approximated as:

3 $$ {a}_p{f}_p\cong {\rho}_e{C}_p\frac{Z^m}{E^n}, $$

where *ρ*_*e*_ is the absolute electron density (e/cm^3^), and *Z* is the effective atomic number. *C*_*p*_ is constant and equals 9.8 × 10^− 24^ [18]. *E* is the energy of the x-ray beam measured in kiloelectron volts. For a numerical fit of the experimental data, *m* is between 3 and 4, and *n* is between 3 and 3.5. In this study, we use *m* = 3.8, *n* = 3.2 [4].

The Compton effect can be approximated with electron density and the total Klein-Nishina cross-section [19]:

4 $$ {a}_c={\rho}_e, $$

5 $$ {f}_c\left(\gamma \right)={C}_0\left\{\frac{1+\gamma }{\gamma^2}\left[\frac{2\left(1+\gamma \right)}{1+2\gamma }-\frac{1}{\gamma}\ln \left(1+2\gamma \right)\right]+\frac{1}{2\gamma}\ln \left(1+2\gamma \right)-\frac{\left(1+3\gamma \right)}{{\left(1+2\gamma \right)}^2}\right\}, $$

6 $$ \gamma =\frac{E}{510.975\ \mathrm{keV}},\kern0.75em {C}_0=2\pi {r}_0^2, $$

where *E* is the x-ray energy and has the unit of kiloelectron volts, and *r*_0_ is the classical electron radius, which equals to 2.818 × 10^− 13^ cm.

If we substitute Eqs. (3)–(5) into Eq. (2), we obtain:

7 $$ \frac{\mu (E)}{\rho}\cong {\rho}_e\left({C}_p\frac{Z^m}{E^n}+{f}_c(E)\right). $$

The two unknown variables *ρ*_*e*_ and *Z* in Eq. (7) can be obtained by the acquisition of two VMIs at distinct energy levels and then analytically solving the resulting set of equations. To maximise the difference between two VMIs and thus improve the accuracy of the solution, we use energy levels at 50 keV and 200 keV. Absolute electron densities can then be converted to relative electron density using known water electron density (3.343 × 10^23^ e/cm^3^).

### Electron density estimation using calibrated conversion function

We used the Gammex phantom to fit a conversion function from HU values measured in two VMIs to relative electron densities. A scan is taken using a relatively high radiation exposure (30 mGy) to acquire almost noise-free calibration images of the phantom at 50 keV and 200 keV. HU values at these two energies are used to fit Saito’s conversion function [14]:

8 $$ {\rho}_e=a\frac{\left(1+\alpha \right)\mathrm{H}{\mathrm{U}}_{\mathrm{H}}-\alpha \mathrm{H}{\mathrm{U}}_{\mathrm{L}}}{1000}+b, $$

where *ρ*_*e*_ is the actual relative electron density taken from the phantom’s data sheet, HU_H_ and HU_L_ are HU values in the VMIs at 50 keV and 200 keV, and *a*, *b*, α are parameters specific to the scanner.

The Gammex phantom consists of twelve materials with known pairs of (*ρ*_*e*_, HU_H_, HU_L_), and there were three unknown parameters (*a*, *b*, *α*) in the equation; this fitting was computed using MATLAB software (v9.2; MathWorks, Natick MA, USA) and a surface-fitting algorithm. The fitting results were then used to compute relative electron densities of the Catphan phantom containing six materials in varied dose scans.

### Error measurement

To describe measurement errors, we computed the percentage error (%Error) as the ratio of the difference of the estimated value (*ρ*_*e*_) to the nominal value of the relative electron density (*ρ*_*n*_):

9 $$ \%\mathrm{Error}=\frac{\rho_e-{\rho}_n\ }{\rho_n}\times 100\%, $$

and *ρ*_*e*_ − *ρ*_*n*_ is noted as absolute error. The overall estimation error is assessed using root mean square error (RMSE) and normalised root mean square error (NRMSE):

10 $$ \mathrm{RMSE}=\sqrt{\frac{1}{N}\sum {\left({\rho}_e-{\rho}_n\right)}^2}; $$

11 $$ \mathrm{NRMSE}=\frac{\mathrm{RMSE}}{\overline{\rho_n}},\kern0.5em \overline{\rho_n}=\left(\frac{1}{N}\sum {\rho}_n\right). $$

To assess correlations between the estimated values and the nominal values, the Pearson correlation *R* was used. In addition, linear regression analysis was performed as fitting:

12 $$ {\rho}_e=\beta \bullet {\rho}_n+\epsilon, $$

where *β* and *ϵ* are regression coefficients (slope and intercept). Coefficient of determination, which describes the goodness of the fit, is noted as *R*^2^. Analysis of covariance (ANCOVA) was performed for the measurement against the group where ${\rho}_e^{\prime }={\rho}_n$, which indicates an ideal measurement. Moreover, a paired *t* test was performed for the measurement against the nominal values.

In order to assess the reproducibility of the estimation and the influence of different radiation exposures, Pearson correlation, RMSE, and NRMSE between measurements and nominal values were computed. A paired *t* test was performed for measurements between the highest dose (30 mGy) and lowest dose (7.5 mGy). All error estimation and statistical analyses were performed using MATLAB software.

## Results

**Fig. 3:**
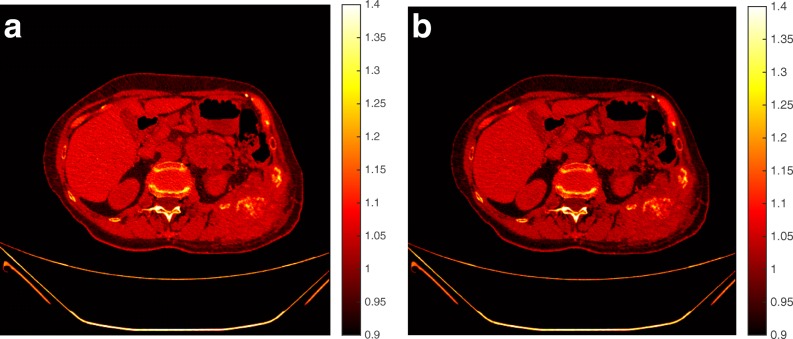
Example of computed electron density in an abdominal scan of a patient. **a** Result from Lehmann’s cross-sectional model. **b** Result from Saito’s conversion function. Scale bar is shown on the right; materials with higher electron density are shown in bright yellow, and lower-density materials appear in dark red

For Lehmann’s cross-sectional model, relative electron densities estimated for the Gammex phantom and the Catphan phantom were highly correlated to the nominal values (*R* = 0.9993, *p* < 0.001) in the scan with a standard dose (20 mGy) (Tables 1 and 2; Fig. 3). Linear regression analysis also suggested that the result was very close to nominal value: with the slope *β* and goodness of fit *R*^2^ very close to 1, and intercept *ϵ* very close to 0: *β* = 1.0028, *ϵ* = 0.0063, *R*^2^ = 0.9986. ANCOVA suggested the measurement had no statistically significant difference from an ideal measurement (*p* = 0.768 for *β*; *p* = 0.557 for *ϵ*). The overall NRMSE was 1.53%. The maximum increase related to water is + 0.021 (cortical bone), and the maximum decrease was − 0.036 (water). If we exclude one of the lung-equivalent inserts (LN 300) and the water insert, all percentage errors are less than 1.79%. The largest percentage error (− 6.82%) was found in the LN-300; owing to the low attenuation of this insert, the small actual electron density (0.264) resulted in larger percentage errors. Nevertheless, the absolute error for LN-300 was − 0.020 and was actually comparable to that of the other inserts. The water insert had the largest absolute error (+ 0.036) compared with all the measurements. A paired *t* test showed the difference between measured electron densities from Lehmann’s model, and nominal values for the Gammex phantom were not statistically significant (*p* = 0.212). The relative electron densities for the Catphan phantom obtained with Lehmann’s model were all higher than their nominal values (*p* = 0.001) (Table 1).

**Table 1:** Electron densities computed from the Lehmann’s cross-sectional model and Saito’s fitted function

				Lehmann		Saito	
			Nominal value	Approximate value	Error (%)	Fitted value	Error (%)
Gammex	1	SB3 cortical bone	1.696	1.675	−1.24%	1.702	+ 0.35%
	2	CB2 50% CaCO_3_	1.471	1.463	−0.54%	1.472	+ 0.07%
	3	CB2 30% CaCO_3_	1.280	1.282	+ 0.16%	1.276	−0.31%
	4	B200 bone mineral	1.109	1.116	+ 0.63%	1.107	−0.18%
	5	IB inner bone	1.107	1.114	+ 0.63%	1.106	−0.09%
	6	LV1 liver	1.062	1.081	+1.79%	1.061	−0.09%
	7	BRN-SR2 brain	1.047	1.064	+1.62%	1.041	−0.57%
	8	*CT water*	*0.990*	*1.026*	*+3.64%*	*1.009*	*+1.92%*
	9	BR-12 breast	0.961	0.977	+1.66%	0.959	−0.21%
	10	AP6 adipose	0.928	0.941	+1.40%	0.923	−0.54%
	11	LN-450 lung	0.466	0.470	+ 0.86%	0.471	+1.07%
	12	*LN-300 lung*	*0.264*	*0.246*	*−6.82%*	*0.255*	*−3.41%*
Catphan	13	Teflon	1.868	1.895	+1.45%	1.850	−0.96%
	14	Delrin	1.363	1.373	+ 0.73%	1.341	−1.61%
	15	Acrylic	1.147	1.161	+1.22%	1.135	−1.05%
	16	Polystyrene	0.998	1.014	+1.60%	0.992	−0.60%
	17	LDPE	0.945	0.959	+1.48%	0.939	−0.63%
	18	PMP	0.853	0.866	+1.52%	0.850	−0.35%
		*Average*	1.086	1.096	1.53%*	1.083	0.87%*

*LDPE* Low-density polyethylene, *PMP* Polymethylpentene

The results for the Gammex phantom (1–12) and the Catphan phantom (13–18) are from scans at 20 mGy. Saito’s fitted function was calibrated using Gammex phantom (*N* = 12) scanned at 30 mGy. ‘Error (%)’ indicates percentage error. An asterisk (*) symbol indicates normalised root mean square error

The italic numbers are the lowest and highest numbers of all the measurement. The underlined numbers are the greatest of the rest in the column

**Table 2:** Measured related electron density at different dose levels

	Dose	7.5 mGy	10 mGy	20 mGy	30 mGy
Lehmann	RMSE	0.0167	0.0170	0.0166	0.0163
	NRMSE	1.54%	1.56%	1.53%	1.5%
	Correlation	0.9992	0.9991	0.9993	0.9993
	Regression, *β*	0.9929	0.9946	1.0028	0.9977
	Regression, *ϵ*	0.0155	0.0120	0.0063	0.0112
Saito	RMSE	0.0104	0.0115	0.0095	0.0102
	NRMSE	0.96%	1.06%	0.87%	0.94%
	Correlation	0.9998	0.9997	0.9997	0.9997
	Regression, *β*	0.9860	0.9886	0.9952	0.9909
	Regression, *ϵ*	0.0099	0.0057	0.0016	0.0058

*RMSE* Root mean square error, *NRMSE* Normalised root mean square error

*N* = 18 for both Gammex and Catphan phantom

**Fig. 4:**
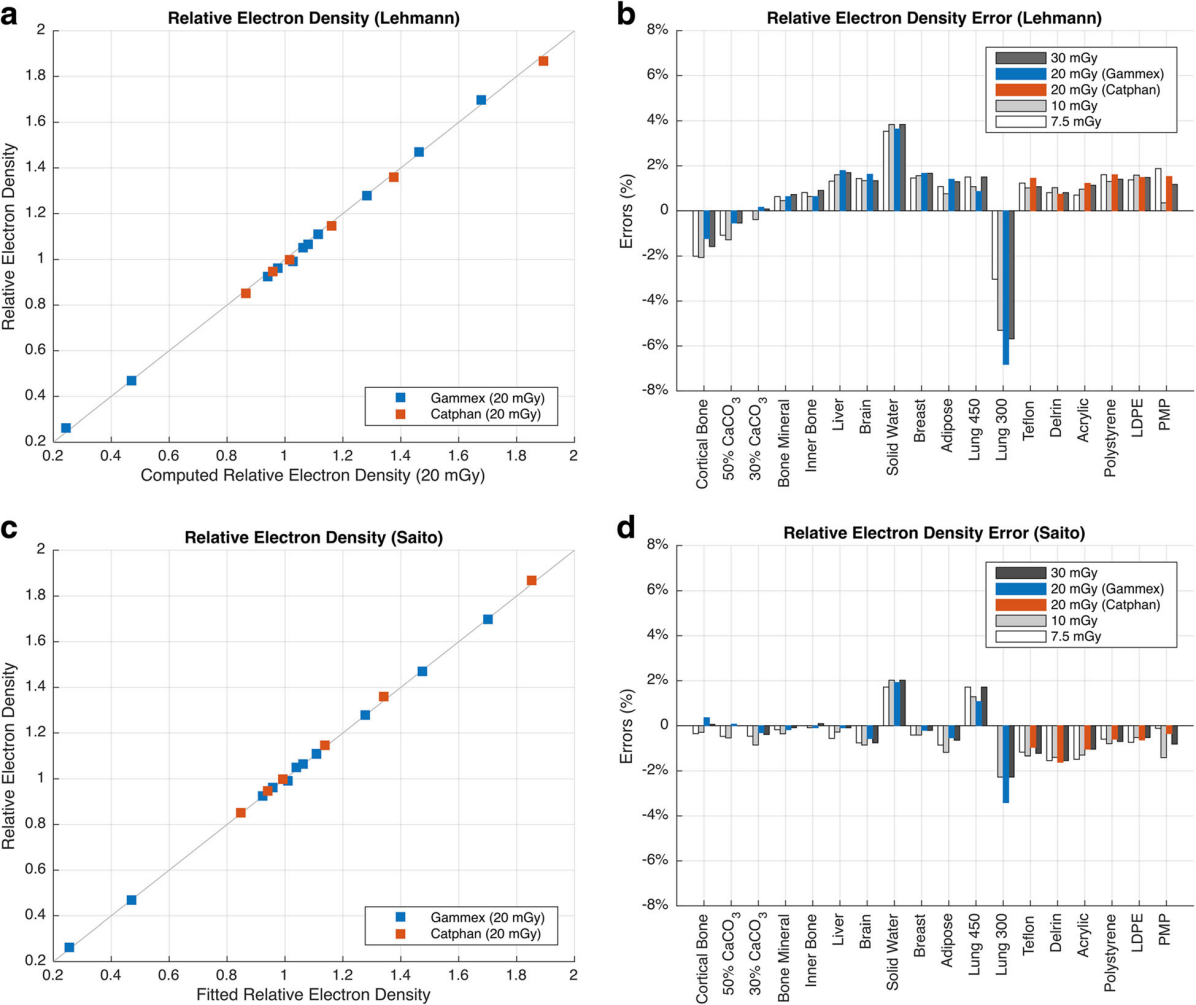
Computed relative electron densities using Lehmann’s cross-sectional model and calibrated relative electron densities using Saito’s conversion function. **a** and **b** Correlation line and percentage error (%Error) between the Lehmann’s cross-sectional model result and the nominal values (*n* = 18). **c** and **d** Corresponding result for Saito’s conversion function result. The diagonal line is drawn in grey in **a** and **c**

For Saito’s calibrated conversion function, the fitting result from a single scan of the Gammex phantom (*n* = 12) at a relatively high radiation dose (30 mGy) was *a* = 0.9704, *b* = 0.9874 and *α* = − 0.02104 for Eq. (8). To validate this fitted conversion function, we computed the relative electron densities for the Gammex and Catphan phantom scans at a standard radiation dose (20 mGy). In Fig. 4, strong correlation to the exact values was observed (*R* = 0.9997, *p* < 0.001). Regression analysis also indicated that the measurements were quite accurate (*β* = 0.9952, *ϵ* = 0.0016, *R*^2^ = 0.9995). ANCOVA also suggested that the measurement showed no significant difference from an ideal measurement (*p* = 0.412 for *β*, *p* = 0.813 for *ϵ*). The overall error was 0.87%. Compared with the results of the fitted cross-sectional model, a higher error was also observed in the case of water (+ 1.92%) and lung inserts (− 3.41%). If we exclude these inserts, the fitted percentage errors are all under 1.61% (*see* Table 1). Similar to the cross-sectional model, the measured electron densities with Saito’s method for the Gammex phantom showed no significant difference from the nominal values (*p* = 0.969). The relative electron densities for the Catphan phantom obtained with Saito’s method were all below the nominal value provided by the manufacturer (*p* = 0.015) (Table 1).

Only subtle influences from different radiation exposures were observed in the measurement. Table 2 shows the RMSE, NRMSE and correlation coefficients between the nominal values and the estimated values for all exposures (30, 20, 10 and 7.5 mGy). The computed relative electron densities between the highest and lowest doses did not show significant changes (*p* = 0.348 for Lehmann’s model, *p* = 0.167 for Saito’s function). On this note, the relatively small effect of different radiation dose levels can also be accounted for by the size of the employed phantoms.

A patient scan applying the relative electron density estimations with Lehmann’s cross-sectional model and Saito’s conversion function is shown in Fig. 3. Both methods have no significant visual differences when depicting tissue with different electron densities.

## Discussion

In this paper, we have illustrated that it is feasible to compute relative electron densities from DLCT acquisitions and that the results for two different phantoms are accurate and reliable. In both methods, we used VMIs while actual x-ray source spectra and detector response information were not required. The measured and nominal values were highly statistically correlated.

For both methods, we observed that a relatively high error appeared in two lung inserts and in the water insert of the Gammex phantom. Previous studies have observed similar inconsistencies with lung inserts and recommended to exclude them [20]. This may be caused by the inhomogeneity of the phantom composition, which is actually thin plastic with small air bubbles as compared with other phantoms based on uniform resin. In our study, higher discrepancy of relative electron density for the water insert was also observed. It is highly likely that in contrast to pure water, which contains mainly hydrogen and oxygen, the material used in the phantom also included certain other substances for solidification. Therefore, manufacturing tolerances may need to be considered. Interestingly, discrepancy in previous studies involving older-generation Gammex phantoms for the brain and adipose phantom was not observed [14].

DECT offers VMIs, which mimic CT images as if the x-ray source is monochromatic. However, real monoenergetic images could be generated only via more advanced image equipment such as a synchrotron [21, 22], which is currently not realistic in a clinical environment. As a result, the cross-sectional model and the VMIs are only approximations. Nevertheless, VMIs estimated from DLCT illustrated much higher accuracy than other DECT solutions [10]. On this note, the application of the cross-sectional model used in DECT was limited to a certain range of atomic numbers of the materials and could introduce errors for hydrogen (*Z* = 1) and higher-density elements (*Z* > 30) [4, 5]. In previous studies with DSCT, the estimation of electron density with images at two energies based on a cross-sectional model was not simple, because it involved a lot of data, such as x-ray beam spectrum and detector corrections. The errors ranged between 1.8% ± 1.6% [5] and 2.3% [6], which are slightly higher than our result (1.53%). The highly correlated result for relative electron density approximation in our study showed that the fitting cross-sectional model is feasible and that the VMIs are highly reliable.

Recently, Almeida et al. [20] investigated the accuracy of relative electron density calculations for DSCT and twin-beam CT, showing high correlations. Similar to our work, Saito’s conversion method was applied for multiple scanners. The investigators observed percentage errors of 1.2% for DSCT and 3.2% for a twin-beam scanner, excluding lung inserts. In contrast to their approach of excluding inhomogeneous lung phantom for calibrating, we also included these materials in our calibration. Nevertheless, in our study we observed that relative electron densities could be measured within 1.61%.

Saito’s conversion function was originally proposed for DSCT with dual-kVp scans. In our study, we applied this method directly to VMIs from DLCT without additional modifications. This conversion method has the advantage that no exact spectral information of the x-ray source is required, meaning that any VMIs in two different energies can be directly applied. According to the original author, a larger spectral separation between the dual-kVp scans led to a smaller *α*. In our case, we used very low and very high VMIs (50 and 200 keV, respectively), instead of two energy scans in DSCT (x-ray source peaks at 80 kV and 140 kV). We found an extremely low *α* (− 0.02104) compared with 0.778 and 0.35 in previous studies [14, 20]. This illustrates the highly competitive spectral performance of the DLCT.

Our study has limitations. First, we did not investigate the effect of different reconstruction methods. We assumed that image filters and advanced iterative reconstruction algorithms can only improve the appearance of the image, but not change the quantitative HU values. Instead, we made scans with different radiation exposures and proved that the methods were still reliable and reproducible across different scans. Second, our study was limited to some degree to the actual phantoms and manufacturing errors needed to be considered. The actual resin-based materials used in the phantom were different from the actual composition of human organs or were insufficient to represent biological materials. Nevertheless, we compared phantoms from two independent manufacturers and showed that the electron density estimation was reliable.

In conclusion, we report an experimental evaluation of relative electron density estimations in DLCT. Our investigation demonstrates that DLCT-based VMIs can be used to estimate relative electron density and that the results are accurate. In the future, DLCT can potentially enhance the workflow of radiation therapy planning by providing spectral data for every scan.

## Abbreviations

ANCOVA: Analysis of covariance
CDTI_vol_: Volume computed tomography dose index
CT: Computed tomography
DECT: Dual-energy computed tomography
DLCT: Dual-layer computed tomography
DSCT: Dual-source computed tomography
HU: Hounsfield units
kVp: Peak kilovoltage
LDPE: Low-density polyethylene
NRMSE: Normalised root mean square error
PMP: Polymethylpentene
RMSE: Root mean squared error
VMI: Virtual monoenergetic image
